# Biofilm production of coagulase-negative staphylococci isolated from rescued wild animals in the Republic of Korea

**DOI:** 10.1186/s13028-019-0485-x

**Published:** 2019-10-24

**Authors:** Sangjun Lee, Jehwi Hwang, Jongwoon Kim, Joonyeop Lee, Hong-Cheul Kim, Haerin Rhim, Jae-Ik Han

**Affiliations:** 1 Laboratory of Wildlife Medicine, College of Veterinary Medicine, Jeonbuk National University, Gobongro 79, Iksan, 54596 Republic of Korea; 2 Jeonbuk Wildlife Center, Jeonbuk National University, Gobongro 79, Iksan, 54596 Republic of Korea

**Keywords:** Methicillin resistance, Reservoir, *Staphylococcus sciuri*

## Abstract

Biofilm production is a well-known causative factor of catheter- and medical device-related sepsis. Its high prevalence in coagulase-negative staphylococci (CoNS) has recently been reported. Information on biofilm production in CoNS isolated from wild animals is lacking. Herein, we studied the biofilm formation capabilities of CoNS isolated from rescued wild animals in the Republic of Korea. Swab samples were collected from the conjunctiva, nasal cavity, perianal area, and rectum for mammals while the sampling was done from the conjunctiva, oral mucosa, pericloacal area, and cloaca for birds. Isolation of CoNS was based on morphological and biochemical analyses along with molecular typing. Biofilm production was analyzed using 96-well plate based quantitative adherence assays. The studies demonstrated that CoNS of mammalian origin have higher biofilm-producing ability (70.4%) than the isolates from birds (62.5%). In particular, all methicillin-resistant (MR) CoNS isolated from mammals were capable of biofilm formation while only 63.3% of MR CoNS isolated from birds could produce biofilms. The MR CoNS isolated from mammals also had a significantly higher ability to form biofilms (100%) than methicillin susceptible CoNS (60.0%) than those isolates from birds. The findings show that wild animals may act as reservoirs as well as possible transmitters of biofilm-mediated antibiotic resistant genes.

## Findings

The pathogenic potential of coagulase-negative staphylococci (CoNS) is well known; however, the potential causes and underlying mechanisms remain unclear. CoNS are nosocomial pathogens in humans, with *Staphylococcus epidermidis* and *Staphylococcus haemolyticus* being the most significant species [1]. They have also been isolated from a variety of farm animals, pets, and wild animals and CoNS are considered as a reservoir of antibiotic resistance genes [2, 3]. Recently, CoNS isolates with high vancomycin resistance were discovered in the saliva of migratory songbirds in the USA [4], suggesting the zoonotic potential of CoNS originating from wild animals. Biofilm formation is a well-known pathogenic characteristic of CoNS that leads to catheter- and medical device-related sepsis [5, 6]. Here we report methicillin resistance prevalence and biofilm formation abilities of CoNS isolated from rescued wild animals in the Republic of Korea.

From December 2016 to February 2017, 120 swab samples were collected from rescued wild animals at the Jeonbuk Wildlife Center. All samplings were done before the animals contacted any medical device or were given any medications. For mammals, samples were collected from the conjunctiva, nasal cavity, perianal area, and rectum, while the sampling was done from the conjunctiva, oral mucosa, pericloacal area, and cloaca of birds. After sampling, the swabs were spread onto trypticase soy agar plates containing 5% sheep blood and were subsequently incubated at 37 °C for 24–48 h. After incubation, CoNS were isolated based on the colony morphology, completion of hemolysis, Gram staining, a conventional catalase test with 5% hydrogen peroxide, coagulase test using EDTA-treated rabbit plasma (BBL Coagulase Plasma, rabbit with EDTA; BD, Sparks, MD, USA), and a DNase test using DNase test agar with methyl green (BD, Sparks, MD, USA). *S. aureus* strain ATCC 25923 (American Type Culture Collection [ATCC], Manassas, VA, USA) and a clinical isolate of *S. epidermidis* confirmed by species-specific polymerase chain reaction (PCR) [7] and sequencing were used as positive and negative controls for coagulase and DNase tests, respectively.

The isolated staphylococci were further identified by 16S ribosomal RNA (16S rRNA) and heat shock protein 60 (*hsp*60) analyses [8–10] (Table 1). After PCR amplification, all amplicons were purified and sequenced. The homology between the deduced nucleotide sequences and a known *S. epidermidis* genomic sequence was analyzed using the BLAST search program (National Center for Biotechnology Information [NCBI], USA). Finally, the species identification of the isolates was confirmed by a multiple-PCR method [11].

**Table 1:** Frequency of coagulase negative staphylococci isolation from wild mammals and birds in the Republic of Korea

	Animal species (scientific name)	Sampling site	*Staphylococcus* sp. (number of isolates)
Mammals (n = 16)	Korean water deer (*Hydropotes inermis*, n = 11)	Conjunctiva	*S. sciuri* (3), *S. delphini* (3), *S. caseolyticus* (1), *S. chromogenes* (1), *S. lentus* (1), *S. warneri* (1)
		Nasal cavity	*S. muscae* (2), *S. chromogenes* (1), *S. haemolyticus* (1), *S. vitulus* (1)
		Perianal	*S. warneri* (1)
		Rectum	*S. hominis* (1), *S. muscae* (1), *S. sciuri* (1)
	Raccoon dog (*Nyctereutes procyonoides*, n = 4)	Conjunctiva	*S. caseolyticus* (2)
		Nasal cavity	*S. caseolytics* (1), *S. sciuri* (1)
		Perianal	*S. haemolyticus* (2)
	Leopard cat (*Prionailurus bengalensis*, n = 1)	Conjunctiva	*S. felis* (1)
		Nasal cavity	*S. felis* (1)
Birds (n = 23)	Black-tailed gull (*Larus crassirostris*, n = 3)	Conjunctiva	*S. epidermidis* (1), *S. sciuri* (1)
		Nasal cavity	*S. sciuri* (4)
		Perianal	*S. haemolytics* (1)
		Cloaca	*S. haemolytics* (1)
	Ring-necked pheasant (*Phasianus colchicus*, n = 1)	Oral mucosa	*S. sciuri* (1)
	Common buzzard (*Buteo buteo*, n = 7)	Conjunctiva	*S. epidermidis* (1), *S. delphini* (1)
		Nasal cavity	*S. sciuri* (3), *S. cohnii* (1), *S. kloosii* (1)
		Oral mucosa	*S. sciuri* (3)
		Perianal	*S. warneri* (7)
		Cloaca	*S. warneri* (4)
	Oriental turtle dove (*Streptopelia orientalis*, n = 2)	Conjunctiva	*S. sciuri* (1)
		Oral mucosa	*S. sciuri* (2)
	Brown hawk-owl (*Ninox scutulata*, n = 6)	Conjunctiva	*S. cohnii* (1), *S. sciuri* (1)
		Nasal cavity	*S. sciuri* (4), *S. cohnii* (2), *S. haemolyticus* (2)
		Perianal	*S. haemolyticus* (1), *S. xylosus* (1)
		Cloaca	*S. epidermidis* (1), *S. haemolyticus* (1), *S. xylosus* (1)
	Tawny owl (*Strix aluco*, n = 2)	Conjunctiva	*S. xylosus* (2)
		Oral mucosa	*S. sciuri* (1), *S. vitulus* (1), *S. xylosus* (1)
	Gray heron (*Ardea cinerea*, n = 1)	Conjunctiva	*S. delphini* (1)
		Oral mucosa	*S. delphini* (1)
	Northern goshawk (*Accipiter gentilis*, n = 1)	Nasal cavity	*S. xylosus* (1)

Methicillin resistance of isolated CoNS was confirmed by the Kirby–Bauer disc diffusion test with a 1 μg oxacillin disc (Oxoid, Hampshire, UK) and a 30-μg cefoxitin disc (Oxoid) and a PCR assay targeting the *mec*A gene [12]. A methicillin resistant (MR) strain (ATCC 25923) and a methicillin susceptible (MS) strain (ATCC 6538) of *S. aureus* were used as controls for these tests.

Biofilm formation ability of the isolated *S. epidermidis* strains was determined through a quantitative adherence assay using 96-well tissue culture plates [13]. Briefly, the isolate from fresh trypticase soy agar with 5% sheep blood was inoculated in trypticase soy broth (TSB) and incubated for 24 h at 37 °C under stationary and aerobic conditions. After incubation, the broth was diluted to the ratio of 1:100 in TSB containing 2% glucose to maximize *ica* operon induction [14]. A total of 200 µL of the cell suspension was subsequently transferred to a U-bottomed 96-well microtiter plates and incubated aerobically for 24 h at 37 °C. The culture was then removed from the wells, and the plates were washed thrice with 200 µL of phosphate-buffered saline to remove non-adherent cells followed by drying of the plates in an inverted position. Adherent biofilms were fixed with 95% ethanol and stained with 100 µL of 1% crystal violet for 5 min. Unbound crystal violet was subsequently removed, and the wells were washed thrice with 300 µL of sterile distilled water. The water was then removed, and the plate was air-dried for 2 h. The optical density (OD) of each well was measured at 570 nm (OD_570_). The analyses were performed in triplicate, and the isolates were classified as strong, moderate, weak, or zero biofilm producers based on their OD_570_ (4 × OD_c_ < OD_570_: strong biofilm producer; 2 × OD_c_ < OD_570_ ≤ 4 × OD_c_: moderate biofilm producer; OD_c_ < OD_570_ ≤ 2 × OD_c_: weak biofilm producer; OD_570_ ≤ OD_c_: no biofilm producer [OD_cutoff_ (OD_c_) = average OD_570_ of negative control + (3 × standard deviation of negative control)]) [15]. *S. aureus* strain ATCC25923 and sterile TSB were used as positive and negative controls, respectively.

From 120 swab samples, 83 CoNS isolates were obtained. Of these, 27 were isolated from 16 wild mammals, while 56 were isolated from 23 wild birds (Table 1). *S. sciuri* was the most commonly isolated species in both mammals and birds. In birds, *S. warneri* was the second most commonly isolated species (n = 11), while *S. caseolyticus* (n = 4) was the second most common species isolated from mammalian samples. Moreover, all *S. sciuri* isolates (n = 5) from mammals were MR, while 18 out of 21 *S. sciuri* (85.7%) samples isolated from birds were MR. Regardless of the origin of the sample, methicillin resistance of CoNS isolates from birds (53.6%) was much higher than that of CoNS isolated from mammals (25.9%).

The biofilm assay results revealed that all CoNS isolated from mammals could form biofilms. This was not associated with methicillin resistance. Among the mammalian MR CoNS, strong or moderate biofilm production was found, while only 60% of mammalian MS CoNS (12/20) were found as strong or moderate biofilm producers. Thus, a significant difference in the prevalence of strong or moderate biofilm production was observed between mammalian-origin MR and MS CoNS (P < 0.001, independent *t* test). This finding differs from observations on MR and MS *Staphylococcus pseudintermedius* isolated from companion dogs in Republic of Korea, indicating no difference of biofilm-producing abilities between MR and MS *S. pseudintermedius* [16]. In birds, 96.4% (54/56) CoNS could form biofilm regardless of methicillin resistance, and 62.5% (35/56) of the CoNS demonstrated strong or moderate biofilm production ability. Table 2 summarizes the results of the biofilm production assay.

**Table 2:** Results of biofilm production assays of 83 coagulase negative staphylococci isolates

Animal	Sample origin	Methicillin resistance (No.)	Mean OD_570_ ± SD	No. (%) of strong or moderate biofilm producer	No. (%) of mild biofilm producer	No. (%) of non-biofilm producer
Mammals	Conjunctive	R (3)	0.426 ± 0.006	3 (100)	0 (0)	0 (0)
		S (10)	0.412 ± 0.032	6 (60.0)	4 (40.0)	0 (0)
	Nasal cavity	R (2)	1.933 ± 0.048	2 (100)	0 (0)	0 (0)
		S (6)	0.134 ± 0.025	2 (33.3)	4 (66.7)	0 (0)
	Perianal	R (1)	0.551 ± 0.050	1 (100)	0 (0)	0 (0)
		S (2)	0.265 ± 0.083	2 (100)	0 (0)	0 (0)
	Rectum	R (1)	0.792 ± 0.028	1 (100)	0 (0)	0 (0)
		S (2)	0.319 ± 0.021	2 (100)	0 (0)	0 (0)
	Total	R (7)	0.926 ± 0.027	7 (100)	0 (0)	0 (0)
		S (20)	0.305 ± 0.028	12 (60.0)	8 (40.0)	0 (0)
Birds	Cloaca	R (0)	–	–	–	–
		S (3)	0.188 ± 0.013	2 (66.7)	1 (33.3)	0 (0)
	Conjunctiva	R (6)	0.253 ± 0.027	3 (50.0)	3 (50.0)	0 (0)
		S (4)	0.284 ± 0.018	3 (75.0)	1 (25.0)	0 (0)
	Nasal cavity	R (15)	0.427 ± 0.096	8 (53.3)	6 (40.0)	1 (6.7)
		S (3)	0.336 ± 0.099	1 (33.3)	1 (33.3)	1 (33.3)
	Oral mucosa	R (5)	1.003 ± 0.512	5 (100)	0 (0)	0 (0)
		S (5)	0.506 ± 0.126	5 (100)	0 (0)	0 (0)
	Pericloacal	R (2)	0.134 ± 0.008	1 (50.0)	1 (50.0)	0 (0)
		S (8)	0.239 ± 0.038	2 (25.0)	6 (75.0)	0 (0)
	Rectum	R (2)	0.479 ± 0.176	2 (100)	0 (0)	0 (0)
		S (3)	1.186 ± 0.407	3 (100)	0 (0)	0 (0)
	Total	R (30)	0.472 ± 0.125	19 (63.3)	10 (33.3)	1 (3.3)
		S (26)	0.412 ± 0.099	16 (61.5)	9 (34.6)	1 (3.8)

*OD* optical density, *SD* standard deviation, *R* resistant, *S* susceptible

Most of the CoNS isolated from wild animals were capable of biofilm production, with 65.1% being classified as either strong or moderate biofilm producers. This data indicates relatively lower prevalence of biofilm producing CoNS in wild animals than in companion animals [16–18]. However, considering that all wild animals evaluated in this study seem to have no prior exposure to antibiotic therapy, this prevalence indicates a wider spread of the biofilm-producing bacteria in the environment. This also implies that subsequent to the acquisition of the antibiotic resistant genes or pathogenic factors by horizontal spread among biofilm-producing staphylococci, wild animals could act as reservoirs and transmitters of these genes or factors [19, 20].

In summary, this study demonstrates the high prevalence of biofilm-producing CoNS in wild animals, indicating the necessity for investigation and management of the wild environment and animals. A future large-scale investigation is necessary to understand and establish effective management strategies.

## Data Availability

The datasets used and/or analyzed during the current study are available from the corresponding author on reasonable request.

## Abbreviations

16S rDNA: 16S ribosomal RNA gene
CoNS: coagulase-negative staphylococci
*Hsp*60: heat shock protein 60
MR: methicillin resistant
MS: methicillin susceptible
OD: optical density
TSB: trypticase soy broth
